# Neural response patterns in inner ear malformations: a scoping review of eABR findings

**DOI:** 10.1007/s00405-026-10257-1

**Published:** 2026-05-05

**Authors:** Sevgi Kadihanoglu, Mehmet Yarali

**Affiliations:** 1 Department of Audiometry, Kutahya Health Sciences University, Kutahya, Türkiye; 2 Department of Audiology PhD Program, Institute of Health Sciences, Hacettepe University, Ankara, Türkiye; 3 Department of Audiology, Hacettepe University, Ankara, Türkiye

**Keywords:** eABR, Inner ear malformations, IP-II, Common cavity, Cochlear nerve

## Abstract

**Purpose:**

This scoping review aimed to synthesize current evidence on electrically evoked auditory brainstem responses (eABRs) in individuals with inner ear malformations (IEMs), with a focus on extraction rates, waveform characteristics, and clinical implications.

**Methods:**

Following PRISMA-ScR and JBI guidelines, a systematic search was conducted in CINAHL, MEDLINE, EMBASE, and PubMed for studies published between January 2005 and June 2025. We included original research articles reporting eABR findings in participants with IEMs. Twenty-one studies met the eligibility criteria.

**Results:**

eABRs were generally obtainable in children with IEMs, though higher thresholds and lower response detectability were frequently observed compared to peers with normal cochlear anatomy. Certain subtypes, such as common cavity and cochlear nerve canal stenosis, often exhibited elevated thresholds. Despite this, wave latency parameters often remained stable. Among patients with Incomplete Partition Type II (IP-II), eABR detectability varied considerably, indicating that cochlear size alone does not determine neural response strength. Factors such as stimulation site, electrode type, and timing of auditory input influenced eABR outcomes.

**Conclusion:**

eABR is a useful tool for evaluating auditory pathway integrity in patients with IEMs. Elevated thresholds may reflect reduced neural synchrony, yet stable waveforms suggest retained function. Incorporating both anatomical and functional data may improve clinical interpretation and decision-making.

## Introduction

Congenital sensorineural hearing loss (SNHL) affects approximately 1 in 1,000 to 1 in 2,000 live births. Inner ear malformations (IEMs) account for 5–15% of congenital SNHL and arise from disruptions in inner ear development during early embryogenesis [1, 2]. To classify these developmental anomalies, several systems have been proposed, with the radiologic classification by Jackler et al. later refined by Sennaroğlu and Saatçi using CT and MRI [3, 4]. This widely adopted framework categorizes IEMs into cochlear, vestibular, semicircular canal (SSC), internal auditory canal (IAC), and vestibular-cochlear aqueduct anomalies, with cochlear malformations further divided into six subtypes: Labyrinthine Aplasia (Michel deformity), Common Cavity (CC), Cochlear Aplasia (CA), Cochlear Hypoplasia (CH), Incomplete Partition Type I (IP-I) and Incomplete Partition Type II (IP-II).

In individuals with inner ear anomalies, the choice between cochlear implant (CI) and auditory brainstem implant (ABI) is guided by audiological evaluations and CT/MRI findings. Assessing cochlear nerve integrity is crucial for predicting implantation outcomes. While imaging supports structural evaluation, functional assessment requires objective measures such as the electrically evoked auditory brainstem response (eABR). As a neurophysiological tool, eABR can be performed preoperatively to assess neural excitability and guide ear selection, and intraoperatively to confirm implant function and auditory nerve integration. During testing, eIII (electrically evoked wave III) and eV (electrically evoked wave V) are recorded, with the eV threshold defined as the lowest current level eliciting this response.

Although eABR is increasingly used in managing patients with IEMs current evidence on its clinical utility remains fragmented and inconclusive. Most studies examine isolated malformation types without cross-comparison, and methodological variability limits the identification of consistent waveform patterns across IEM subtypes. While some findings support eABR as a marker of cochlear nerve integrity and a tool for distinguishing CI vs. ABI candidacy, others report inconsistent results, creating interpretive challenges. This scoping review aims to synthesize existing literature on eABR in IEM, clarify its clinical and surgical applications, and highlight neural response patterns to guide future research and improve clinical decision-making.

## Materials and methods

This scoping review was conducted to synthesize the current evidence on eABR findings in individuals with IEMs. The methodology was guided by the Joanna Briggs Institute (JBI) Manual for Evidence Synthesis for scoping reviews, and the reporting followed the PRISMA Extension for Scoping Reviews (PRISMA-ScR) checklist.

## Search strategy

The authors independently conducted a literature search across CINAHL, MEDLINE, EMBASE, and PubMed, covering studies from January 2005 to June 2025. The time frame from January 2005 to June 2025 was selected to capture evidence generated after the widespread clinical adoption of cochlear implantation and the more standardized use of eABRs in both intraoperative and postoperative settings. Earlier studies primarily focused on experimental or exploratory electrophysiological techniques and lacked consistent reporting of eABR parameters relevant to current clinical practice. The strategy combined controlled vocabulary and free-text terms related to eABR and IEM subtypes (e.g., “eABR inner ear”, “eABR Mondini malformation”, “IP-II eABR”, “CND eABR”). Search strings were adapted for each database. Reference lists of included studies were also manually screened for additional eligible articles.

### Eligibility criteria

Studies were included if they met the following criteria:

- Original research articles with experimental or observational design (e.g., retrospective or prospective studies).
- Participants with any type of IEMs.
- Presence of the cochlear nerve (normal, hypoplastic, or thin).
- Use of intraoperative eABR or eABR as a measure of neural integrity.
- Articles published in English.

No age restrictions were applied.

### Exclusion criteria:

- Articles not published in English.
- Studies involving participants with complete cochlear nerve aplasia.
- Studies that didn’t report eABR results separately for individuals with IEMs.
- Review articles, editorials, conference abstracts, clinical trials or case reports.
- Studies reporting single-case observations or very small case series that did not allow extraction of interpretable eABR patterns at the malformation subtype level.

Although scoping reviews do not aim to formally appraise study quality, studies limited to isolated case descriptions or extremely small case series were excluded because they did not permit meaningful synthesis of eABR characteristics across inner ear malformation subtypes. Clinical trials were excluded because the primary objective of this scoping review was to summarize observational and clinically descriptive evidence on eABR characteristics, rather than intervention efficacy or treatment outcomes. Many clinical trials focus on therapeutic protocols or comparative interventions, where eABR is not reported as a primary or detailed neurophysiological outcome. Although no age restrictions were applied, the included literature was predominantly pediatric. This reflects the clinical reality that eABR is most frequently utilized during early implantation periods, particularly in children with congenital inner ear malformations. Therefore, the pediatric predominance of the evidence was considered appropriate for addressing the review objectives.

## Study selection

The study selection process was conducted in two phases. Initially, records identified through database searches and manual reference screening were assessed for eligibility. After the removal of 102 duplicates, a total of 258 unique records were screened based on titles and abstracts. Of these, 232 records were excluded for not meeting the inclusion criteria.

The remaining 26 full-text articles were reviewed in detail. Of these, 5 studies were excluded for the following reasons:

- Not published in English (*n* = 2, e.g., Wang, Wei [5], Xia and Zhang [6])
- Clinical trial design (*n* = 1, e.g., Cushing, Papsin [7])
- Aim not aligned with the review objective (*n* = 1 e.g., Qiao, Li [8])
- Insufficient sample size (*n* = 1, e.g., Lundin, Stillesjö [9])

**Fig. 1:**
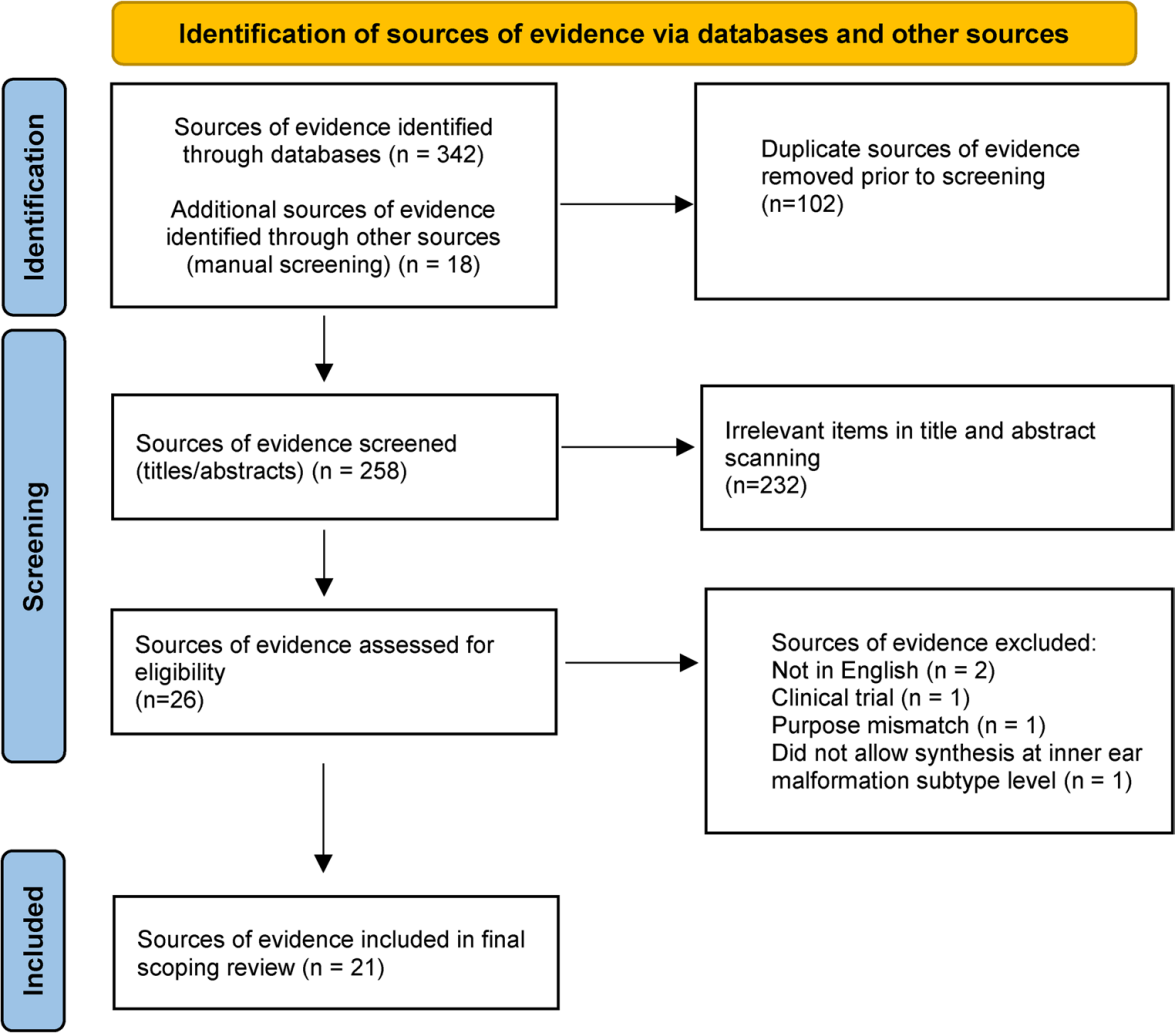
PRISMA-ScR flow diagram of study selection

Ultimately, 21 original research articles met the inclusion criteria and were included in the final synthesis. The entire selection process is illustrated in Fig. 1, based on the PRISMA-ScR flow diagram.

### Data extraction and synthesis

A standardized data extraction form was developed. The following data were extracted from each included study:

- Author(s), year, and country.
- Study design and sample size.
- Type of IEM.
- Type of electrode used (CI, ABI, test electrode).
- eABR parameters (e.g., presence/absence of waves, latencies, waveform morphology).
- Clinical or surgical implications derived from eABR findings.

Due to the heterogeneity in study designs and reported outcomes, a descriptive synthesis approach was used. Results were grouped under thematic categories related to eABR interpretation and its relevance to clinical decision-making. Intraoperative or postoperative eABR recordings were included in all articles.

## Results

Table 1 provides a summary of the included studies, outlining participant characteristics, IEM subtypes, eABR methodologies, and key findings relevant to auditory pathway assessment.

**Table 1:** Summary of included studies reporting eABR findings in children with IEMs

Author (year)	Country	Sample size / age group	IEM type	eABR method	Key findings
Chen et al. (2023)	China	*n* = 40 / Children	Normal; CH; IP-II; EVA	Intraoperative	eABR recorded in children prior to CI
Cinar et al. (2011)	Turkey	*n* = 35 / Children	Normal; IP-I; IP-II; LVAS	Intraoperative	Objective test techniques evaluated in CI users with IEM
Cinar et al. (2017)	Turkey	*n* = 11 / Not specified	Normal, IP-I, IP-II, CC, CH; CNCs-CND	ITE and CI/ABI electrodes	eABR compared between ITE and implanted devices
Di Stadio et al. (2019)	Italy	*n* = 26 / Children	Normal; IP-II	Postoperative	Compared eABR in patients with IP-II and normal anatomy
Kaga et al. (2020)	Japan	*n* = 18 / Infants	Normal; CC	Intraoperative	eABR and developmental outcomes examined in CC cases
Kim et al. (2008)	USA	*n* = 39 / Children	IP; CC; narrow IAC	Preoperative	eABR’s role in CI candidacy in IEM children
Luo et al. (2024)	China	*n* = 26 / Children	Normal; CNCs	Intraoperative or immediate postoperative	eABR examined in CNCs patients
Minami et al. (2015)	Japan	*n* = 38 / Children	Normal; CC; IP-I; CH-III; EVA; CNCs	Intraoperative	Evaluated eABR use during CI in IEM children
Minami et al. (2021)	Japan	*n* = 60 / Not specified	Modiolus deficiency and/or IAC CND	Intracochlear	eABR findings according to modiolus deficiency and/or IAC CND status
Qiao et al. (2017)	China	*n* = 88 / Children	Normal; LVA; CC; SSC malformations; IAC stenosis	Postoperative	Objective tests post-CI; focus not primarily eABR
Song et al. (2010)	Korea	*n* = 13 / Not specified	CNCs	Postoperative	eABR obtaining results in CNCs
Wang et al. (2023)	China	*n* = 74 / Not specified	Normal; CND	Preoperative	Evaluated auditory pathways by eABR
Wang et al. (2018)	China	*n* = 16 / Children	Normal; CNCs	Postoperative	eABR characteristics in CNCs patients
Yamazaki et al. (2015)	Japan	*n* = 19 / Not specified	CND	Intraoperative	MRI and eABR used to predict CI outcomes
Yamazaki et al. (2024)	Japan	*n* = 179 ears / Children	Normal; CC; IP-I; IP-II; CND	Intraoperative	Intraoperative eABR predicts optimized stimulation
Yamazaki et al. (2014)	Japan	*n* = 5 / Children	CC	Intraoperative	Spatial distribution of neurons via eABR in CC patients
Yang & Liu (2017)	China	*n* = 47 / Not specified	Normal; LVAS; IP-II; IAC stenosis	Intraoperative	eABR characteristics in patients with inner ear malformations
Zhang et al. (2025)	China	*n* = 112 / Children	Various IEMs	Intraoperative	eABR used to guide ABI placement
Zhu et al. (2022a)	China	*n* = 58 / Not specified	Normal; IP-II	Intraoperative	eABR characteristics in IP-II patients
Zhu et al. (2022b)	China	*n* = 54 / Children	Normal; EVA	Postoperative	eABR features in children with EVA
Zhu et al. (2023)	China	*n* = 75 / Children	Normal; IP-II	Intraoperative	Effect of cochlear size on eABR

*eABR *electrically evoked auditory brainstem response, *CC* common cavity, *CH* cochlear hypoplasia, *IP* incomplete partition, *EVA *enlarged vestibular aquaductus, *LVAS *large vestibular aquaductus syndrome, *IAC* internal acoustic canal, *CNCs *cochlear nerve canal stenosis, *CND *cochlear nerve deficiency, *SSC *superior semisircular canal,  *MRI* magnetic resonance imaging, *ABI* auditory brainstem implant, *CI* cochlear implant, *ITE* intracochlear test electrode, *IEM* inner ear malformation

### eABR detectability in children with and without IEMs

Across multiple studies, eABR responses were generally obtainable in children with IEMs; however, response quality and robustness were consistently lower than those observed in peers with normal cochlear anatomy. Detection rates in IEM ears showed substantial variability, ranging from approximately 33% to 90%, whereas anatomically normal ears almost uniformly demonstrated clear and reproducible waveforms [10–13]. Although overall response detectability was frequently high, children with specific anomalies, including cochlear hypoplasia (CH) and (en)larged vestibular aqueducts (EVA/LVA), more often exhibited reduced proportions of ‘Good’ responses in conjunction with elevated stimulation thresholds.

This pattern was further supported by intraoperative recordings. Studies employing round window niche or round window membrane stimulation consistently reported higher eABR thresholds in IEM groups, while eIII and eV latencies largely remained comparable to control ears [14]. Similarly, comparisons across different stimulation pulse widths demonstrated persistently higher thresholds and lower eV extraction rates in malformed ears, despite preserved waveform timing [15].

### Influence of recording technique and stimulation site on eABR outcomes

Several studies examined the influence of recording technique and stimulation site on eABR outcomes in children with complex IEMs. In this context, the use of an intracochlear test electrode (ITE) was shown to aid differentiation between candidates for cochlear implantation and those requiring an ABI [16]. When cochlear aperture and auditory nerve integrity were preserved, as in cases of IP-II or CH, eABR waveforms recorded via ITE closely resembled those obtained using conventional CI electrodes. By contrast, no eABR responses were elicited in ears with cochlear aperture aplasia or severe hypoplasia, despite increased stimulation intensity. In these cases, ABI stimulation produced reliable waveforms, confirming intact brainstem responses. Notably, no eABR responses were observed in the reported common cavity (CC) case, indicating limited cochlear neural tissue despite preserved central conduction pathways.

### Threshold and latency characteristics in EVA/LVA and IP-II

Across studies examining EVA and IP-II, eABR outcomes demonstrated relatively preserved waveform timing with variable response detectability and threshold behavior. In EVA, thresholds were generally lower at apical and mid-cochlear stimulation sites, whereas greater variability was observed with basal stimulation. Differences in eIII latencies between EVA and anatomically normal ears were not statistically significant, and eV latency differences were limited to the most apical electrode position [17].

In IP-II, eABR recordings obtained via round window niche and round window membrane stimulation showed reduced response detectability compared with control ears (approximately 52–56% vs. ~81%), while thresholds and eIII/eV latencies were largely comparable across groups [18]. Longitudinal assessments further demonstrated stable eIII and eV latencies over time in both IP-II and control ears, although lower eV amplitudes were consistently observed in the IP-II group at surgery and at six-month follow-up [19]. Analyses incorporating cochlear morphology reported associations between smaller cochlear dimensions and prolonged response latencies in both control and IP-II cohorts, with similar directional patterns observed across groups [20].

### Threshold elevation and reduced detectability in severe inner ear malformations (CC, CNCs, CND)

Across studies focusing on severe IEMs, including CC, cochlear nerve canal stenosis (CNCs; a bony canal abnormality), and cochlear nerve deficiency (CND; nerve hypoplasia or absence), eABR outcomes were characterized by reduced response detectability and elevated stimulation thresholds compared with ears with normal cochlear anatomy. Across these malformation groups, response rates were consistently lower, often ranging between approximately 50% and 80%, with the lowest detectability reported in CND and CNCs cohorts [21, 22]. Threshold elevations were observed across stimulation sites, particularly at mid- and basal-electrode positions, whereas eV latencies generally remained comparable to control ears when responses were present [21–23].

Within CC malformations, eABR responses were most reliably elicited from electrode positions along the anteroinferior inner wall of the cavity, with reported mean eV latencies of approximately 4.0 ms [24, 25]. In malformations involving the cochlear nerve, response detectability was more variable. In CNCs, reduced response rates were accompanied by higher thresholds and, in some studies, prolonged eV latencies. Similarly, in CND, eABR recordings frequently demonstrated elevated eV thresholds and reduced response amplitudes, with absent responses reported in a subset of cases despite increased stimulation intensity [13].

Imaging–electrophysiological comparisons further demonstrated variability in eABR outcomes according to cochlear nerve morphology. Positive eABR responses were more frequently observed when the vestibulocochlear nerve (CN8) diameter was equal to or larger than that of the facial nerve (CN7) than when the facial nerve exceeded the vestibulocochlear nerve in size [23].

### Comparative eABR outcomes across IEM subgroups

Distinct eABR response profiles have been observed across different IEM types. When IEMs were categorized into three subgroups—Group 1: IP (*n* = 11), Group 2: CC (*n* = 20), and Group 3: narrow IAC (*n* = 8)—a statistically significant difference in eV amplitude was reported among them. The narrow IAC group demonstrated markedly lower eV amplitudes and longer latencies compared to both IP and CC groups. No other significant differences were found among the three subgroups [26].

In a larger retrospective series, eABR responses were evaluated in 60 patients with IEMs, grouped according to the presence of the modiolus and the integrity of the cochlear nerve. Four stages were defined: Stage I (modiolus + / normal CN, *n* = 23), Stage II (modiolus – / normal CN, *n* = 13), Stage III (modiolus + / IAC CND, *n* = 20), and Stage IV (modiolus – / IAC CND, *n* = 4). Typical eABR waveforms were obtained in 93% of Stage I, 36% of Stage II, 23% of Stage III, and 50% of Stage IV cases, with response rates significantly higher in Stage I than in all other groups [27].

Comparative analysis of pediatric CI users with IEMs revealed that thresholds and eV latencies varied according to malformation type. When IP- I (IP-I), IP-II (IP-II) and control groups were compared, the IP-I group exhibited higher thresholds and longer latencies than the IP-II group. In both IP subgroups, thresholds were higher at basal than apical stimulation sites, and IP-II results more closely resembled those of the control group. Latency differences across electrode positions were not reported for either IP subtype [12].

Intraoperative monitoring in children with IEMs further demonstrated that eABR thresholds could be used to predict postoperative current requirements. Among 79 malformed ears, eV was identified in 73% overall, with detection rates of 81.8% in CC, 85.0% in IP-I, 100% in IP-II, and 54.8% in CND. Three CND ears (3.8%) showed no responses despite increased stimulation intensity. Mean eV thresholds were highest in CC and CND groups and lowest in IP-I and IP-II. A strong correlation was found between eV thresholds and maximum comfortable current (cC) levels, particularly in CC and CND cases [28].

Prospective assessment of 112 children with profound hearing loss and severe IEMs undergoing ABI revealed that eABR yield varied substantially by malformation type. The mean proportion of eABR-positive electrodes was 72.73% ± 17.99%, with higher rates in children with CH, CC, or CA than in those with more severe anomalies such as Michel deformity or complete CND. At device activation, eABR positivity remained greater in CH, CC, and CA (~ 67%) compared with IP-II or complete CND (~ 52%) [29].

To facilitate interpretation, Table 2 provides a comparative summary of eABR outcomes across malformation subtypes, highlighting overall patterns of detectability, thresholds, latencies, and clinical implications.

**Table 2:** A comparative table across malformation types

IEM type	eABR detectability	Thresholds	Latency	Clinical implications
IP-II	High (often similar to normal) but variable across studies	Normal to slightly high	Stable	Reliable indicator for CI candidacy
CC	Variable, sometimes low	Significantly high	Near normal	CI possible if CN8 intact; helps optimize electrode placement
CH	Variable; often detectable but weaker	Slightly higher	Stable or slightly prolonged	May benefit from CI if cochlear nerve intact; outcomes variable
EVA / LVAS	High, close to normal	Comparable to normal	Stable	Good prognosis; useful for predicting speech/hearing outcomes
CNCs	Reduced (~ 50%)	Higher	Prolonged	May indicate fewer SG cells apically; ABI considered if CI fails
CND	Very low or absent	High	Abnormal	Limited reliability; MRI + eABR together guide CI vs. ABI
Narrow IAC	Reduced	Elevated	Prolonged	Reflects thinner auditory nerve; outcomes less predictable

*eABR *electrically evoked auditory brainstem response, *CC* common cavity, *IP* incomplete partition, *EVA* enlarged vestibular aquaductus, *LVAS *large vestibular aquaductus syndrome, *IAC* internal acoustic canal, *CNCs* cochlear nerve canal stenosis, *CND* cochlear nerve deficiency, *MRI* magnetic resonance imaging, *ABI *auditory brainstem implant, *CI* cochlear implant,  *IEM *inner ear malformation, *SG* spiral ganglion, *CN8* vestibulocochlear nerve, *CH *cochlear hypoplasia

## Discussion

This scoping review provides a comprehensive overview of eABR findings in individuals with IEMs. It summarizes the reported rates of eABR detectability, characteristic waveform patterns, and differences observed both between normal and malformed ears and across specific IEM subtypes.

### Methodological considerations in eABR recording

eABRs have been recorded in preoperative, intraoperative, and postoperative settings, each providing complementary insights into auditory pathway integrity (Table 1). Pre- and postoperative protocols generally follow standardized procedures, whereas intraoperative approaches exhibit greater variability across studies. Needle electrodes are typically preferred to minimize electrical artifacts, and alternative placements tested in several investigations did not significantly influence waveform morphology or latency outcomes [18, 24]. The transtympanic round window (tt-eABR) method has also been attempted; however, it was associated with considerable artifact contamination and response instability [26, 30]. To overcome these limitations, the use of an intracochlear test electrode (ITE) was introduced, offering improved artifact control and more accurate simulation of CI stimulation. Nonetheless, clear eV responses were not consistently obtained in cases with structural deformities or CND [16]. These methodological observations highlight the importance of standardizing intraoperative stimulation and recording protocols to improve inter-study comparability and clinical interpretation of eABR data. In addition, future studies should examine how specific morphological anomalies influence eABR detectability and waveform quality.

Interpretation of eABR findings across studies is complicated by substantial methodological heterogeneity. Included studies differed widely in electrode type (CI electrode, intracochlear test electrode, ABI), stimulation modes, recording environments (preoperative, intraoperative, postoperative), and criteria used to define waveform presence, amplitude, and latency. In addition, the absence of standardized definitions for eABR detectability and threshold limits direct quantitative comparison across malformation subtypes. This methodological variability is particularly relevant in malformed cochleae, where electrode–neural interface characteristics may differ markedly from normal anatomy.

### Diagnostic value of eABR compared to eCAP

Both eABR and electrically evoked compound action potentials (eCAP) serve as key indicators of auditory nerve and pathway integrity [31]. However, eCAP primarily reflects activity at the cochlear level, whereas eABR assesses the neural synchrony of the entire auditory pathway up to the brainstem. In individuals with IEMs, eCAP responses may not always be elicited due to disrupted peripheral excitation, while eABR can still be successfully recorded in most cases [10, 12]. This distinction underscores the clinical value of eABR as a complementary and sometimes superior measure for confirming auditory pathway continuity when eCAP is absent. Incorporating both responses into pre- and intraoperative assessments could therefore improve diagnostic confidence and assist in differentiating candidates likely to benefit from cochlear versus brainstem implantation.

### eABR findings in specific malformation subtypes

Obtaining eABR in EVA demonstrates that the postoperative auditory conduction pathway resembles that of patients with normal inner ear anatomy. Moreover, the correlation between vestibular aqueduct (VA) width and eABR wave latencies suggests that VA measurements can serve as an indicator of auditory pathway status in affected children. Using eABR in the postoperative period may therefore provide valuable insight into the maturation of auditory pathways and help predict subsequent speech and hearing development [17].

In IP-II malformations, eABR could not be obtained in a subset of patients when stimulated through the RWN or RWM—a finding likely related to cochlear structural deformities and reduced spiral ganglion cell populations. These responses often presented as either fully present or entirely absent, supporting the need for individualized, objective assessment [18]. Consistent with these findings, Di Stadio et al. [19] reported lower eV amplitudes in IP-II compared to normal anatomy, attributing this to maturational differences of the brain or a limited number of spiral ganglion cells in the modiolus. The absence of intergroup differences in eIII latency may relate to surgical factors, as appropriate electrode insertion can sufficiently stimulate more proximal neural elements to elicit eIII but not eV. Clinically, these findings indicate that eABR may help identify IP-II patients requiring modified mapping strategies due to reduced brainstem response strength.

According to Kaga et al. [24], obtaining eABR in infants with CC malformation suggests potential benefit from CI if CN8 is well developed. Preserved eV latencies indicate intact brainstem conduction, whereas nearly doubled thresholds compared to controls may reflect reduced cochlear neuron numbers. Thus, eABR can help estimate neuronal density and pathway integrity. Supporting this, Yamazaki et al. [25] showed that auditory neurons cluster mainly along the anteroinferior inner wall of the cavity and emphasized the role of eABR in guiding electrode placement and programming in CC cases.

Wang et al. [32], reported that the eABR detection rate in children with CNCs was about half that of controls, reflecting underdeveloped nerves and incomplete conduction. In normal anatomy, thresholds were lowest at basal sites due to proximity to the modiolus, but this advantage was absent in CNCs, suggesting fewer apical spiral ganglion cells. From a clinical perspective, cochlear implantation is not contraindicated in these patients; however, when preoperative or intraoperative assessments reveal poor eABR responses and limited auditory benefit, ABI should be considered as an alternative [21, 26].

Wang et al. [13] emphasized that the auditory pathway from the spiral ganglion cells to the cochlear nerve may be significantly weaker in the cochlear nerve hypoplasia group than in the normal anatomy group. Notably, eABR responses may still be elicited even when imaging fails to visualize the cochlear nerve, possibly due to fusion of the auditory and facial or vestibular nerves. This presents a critical clinical dilemma: radiologic findings may suggest cochlear nerve deficiency and favor consideration of an ABI, whereas the presence of an eABR response indicates functional auditory pathway activation that may support proceeding with CI. Therefore, eABR serves as a crucial complementary diagnostic tool in cases with inconclusive imaging, helping to refine the decision-making process between CI and ABI candidacy [13, 26].

### Clinical utility of eABR in surgical planning

Individuals with cochlear nerve anomalies may also face a dilemma regarding the use of CI or ABI. Yamazaki et al. [23] proposed that when the diameter of CN7 exceeds that of CN8 and no appropriate eV responses can be obtained in eABR, ABI should be considered; otherwise, CI remains a viable option. Importantly, they also reported postoperative categories of auditory performance (CAP) outcomes, showing that children with CN7 equal to or larger than CN8 and positive eABR achieved CAP scores of 3 or higher, whereas those with CN7 smaller than CN8 and negative eABR responses remained at CAP scores of 3 or lower. These findings demonstrate that reporting postoperative audiological outcomes is essential to confirm whether preoperative and intraoperative criteria truly predict long-term benefit. Similarly, Cinar et al. [16] stated that the presence of eV in eABR supports CI candidacy, whereas absent waves require further consideration of preoperative audiological and radiological findings. If behavioral tests are positive and the auditory nerve is visible on imaging, CI may still be pursued; otherwise, ABI should be considered. However, they did not provide systematic postoperative audiological outcomes, which limits the strength of their conclusions. Future research incorporating standardized postoperative outcome reporting and multimodal preoperative evaluation (behavioral, radiologic, and electrophysiologic) will be essential to establish evidence-based surgical guidelines for patients with auditory nerve anomalies. Ultimately, combined electrophysiological and radiological findings can therefore assist in this complex decision-making process.

### Variability across and within IEM types

Table 2 summarizes the subtype-specific variability in eABR outcomes, illustrating consistent patterns in detectability, thresholds, and latencies across different IEMs. Among these subtypes, children with narrow IAC typically exhibit longer eV latencies and lower amplitudes than those with incomplete partition (IP) or CC malformations, findings likely associated with a thinner or hypoplastic auditory nerve and reduced neural transmission capacity. Within the IP group, IP-II results tend to resemble normal cochlear anatomy more closely than IP-I, a difference attributed to the degree of cochlear structural preservation [12]. In cases with both IAC CND and modiolus deficiency, the likelihood of obtaining typical eABR waves was unexpectedly higher in those with modiolus deficiency than in those with preserved modiolus The higher percentage of eABR detectability in cases with modiolus deficiency was not explained by the researchers [27]. The reason for this situation may be due to the difference in the distribution of the number of participants. 20 of the 60 patients were classified as Stage III, and 4 as Stage IV. In comparison, while 2 individuals in Stage IV would be classified as 50% to show typical eABR, this situation would differ in Stage III. To address this limitation, future studies should include more balanced sample sizes when comparing subgroups.

These findings highlight the complex relationship between IEMs and eABR outcomes. While anomalies often lead to elevated thresholds—suggesting reduced neural synchrony—latencies remain relatively stable. Conditions like Mondini deformity and IAC stenosis may require higher stimulation for reliable responses, emphasizing the need for individualized programming. Despite reduced waveform detectability with greater anatomical severity, threshold and latency values remain statistically similar, especially in IP-II, indicating notable interindividual variability. Thus, cochlear morphology alone may not determine neural responsiveness; integrating anatomical, physiological, and experiential factors could improve eABR interpretation and guide personalized CI strategies.

From a clinical perspective, these findings support the role of eABR as an objective, patient-specific tool for assessing auditory pathway integrity and guiding intraoperative monitoring, electrode placement, and postoperative programming in individuals with complex inner ear malformations. Future studies integrating quantitative imaging markers with electrophysiological and behavioral outcomes will be crucial to establish predictive models linking structural anomalies to functional hearing performance and to develop evidence-based, patient-specific rehabilitation protocols.

### Limitations

This review did not apply an age limit for eABR recording. Enomoto et al. [33], found no significant age-related differences in eV latency across 58 participants (infants, children, adults, elderly) with varied auditory profiles. However, no study specifically examined age-related changes in individuals with IEMs.

The pediatric predominance of the included studies may limit generalizability to adult populations; however, this distribution reflects current clinical practice in which eABR is primarily applied during early auditory rehabilitation.

In addition, although this review did not formally assess study quality, most included studies were heterogeneous, involved small samples, and showed methodological variability, which should be considered when interpreting the findings.

## Conclusion

eABR is a valuable tool for assessing auditory pathways up to the brainstem and is often more applicable than eCAP. Clinically, eABR appears most reliable in IP-II and EVA, where findings often resemble those of normal anatomy, while responses are more variable in CC and markedly reduced in cases of CNCs or deficiency. These limitations underscore the need to integrate eABR with detailed imaging and behavioral assessments. Overall, combining anatomical, physiological, and functional data may provide the most accurate guidance for CI versus ABI candidacy and for individualized programming strategies. 

## Data Availability

No template data collection forms, extracted data, or analytic code are publicly available for this review. All relevant data extracted from the included studies are reported within the manuscript and its supplementary materials.
